# Peer Review Evaluation Process of Marie Curie Actions under EU’s Seventh Framework Programme for Research

**DOI:** 10.1371/journal.pone.0130753

**Published:** 2015-06-30

**Authors:** David G. Pina, Darko Hren, Ana Marušić

**Affiliations:** 1 Research Executive Agency, European Commission, Brussels, Belgium; 2 Department of Psychology, School of Humanities and Social Sciences, University of Split, Split, Croatia; 3 Department of Research in Biomedicine and Health, School of Medicine, University of Split, Split, Croatia; Max Planck Society, GERMANY

## Abstract

We analysed the peer review of grant proposals under Marie Curie Actions, a major EU research funding instrument, which involves two steps: an independent assessment (Individual Evaluation Report, IER) performed remotely by 3 raters, and a consensus opinion reached during a meeting by the same raters (Consensus Report, CR). For 24,897 proposals evaluated from 2007 to 2013, the association between average IER and CR scores was very high across different panels, grant calls and years. Median average deviation (AD) index, used as a measure of inter-rater agreement, was 5.4 points on a 0-100 scale (interquartile range 3.4-8.3), overall, demonstrating a good general agreement among raters. For proposals where one rater disagreed with the other two raters (n=1424; 5.7%), or where all 3 raters disagreed (n=2075; 8.3%), the average IER and CR scores were still highly associated. Disagreement was more frequent for proposals from Economics/Social Sciences and Humanities panels. Greater disagreement was observed for proposals with lower average IER scores. CR scores for proposals with initial disagreement were also significantly lower. Proposals with a large absolute difference between the average IER and CR scores (≥10 points; n=368, 1.5%) generally had lower CR scores. An inter-correlation matrix of individual raters' scores of evaluation criteria of proposals indicated that these scores were, in general, a reflection of raters’ overall scores. Our analysis demonstrated a good internal consistency and general high agreement among raters. Consensus meetings appear to be relevant for particular panels and subsets of proposals with large differences among raters’ scores.

## Introduction

Peer review plays a central role in evaluating scientific research, either when it is communicated in scientific journals [1,2] or when it is submitted to granting bodies [3]. While peer review in journals has been widely studied and has emerged as a separate research discipline [4], the knowledge about peer review of grant applications is relatively scarce [5,6]. A 2007 Cochrane systematic review which addressed the effectiveness of the grant peer review process demonstrated little empirical evidence for the effects of grant peer review [3]. Recent qualitative and quantitative research of grant peer review at institutional, funding agency or national levels identified the variability of the expert reviewers (raters) assessment criteria as an important aspect of the grant review process either within the same [7–17] or between different [18] review systems. Some studies have reported differences between grant reviews in different research disciplines, with greater agreement between raters in humanities compared to other fields [13]. Whereas some studies did not find advantages of panel discussions to individual raters in improving the reliability of grant peer review [8,14], others demonstrated practical value of panel meetings for a subset of grant applications [19]. Except the study of Mutz et al [13], which used more than 8 thousand proposals from the Austrian Science Fund to model inter-rater reliabilities, other observational studies into the reliability of grant peer review used much smaller numbers, ranging from about 30 [12] to more than 800 proposals [14,20].

We investigated applications to one of the largest research granting schemes of the European Union—the “Marie Curie Actions” (MCA—the acronym is used here to refer to all different Marie Curie-related actions, programs and fellowships) within the EU’s Seventh Framework Programme for Research and Technological Development (FP7). Since 1996, “Marie Curie” has become synonymous with EU funding dedicated to the mobility, capital enhancement and potential improvement of research human resources. Over time, the MCA gained reputation among the research community, and is today one of the most popular and well-respected EU research funding programmes. Conceived to support training, international mobility and career development of researchers within and beyond Europe, the MCA budget has grown with successive research Framework Programs and has typically represented 8–10% of its total funding.

In the FP7 context, the MCA budget amounted to 4750 million Euro (for the 2007–2013 period) [21], and comprised a set of different schemes, each targeting a specific audience and addressing a particular aspect of the policy objectives detailed in the ‘People’ Specific Programme [22]. Among those schemes, the ‘Initial Training Networks’ (ITN) was the main instrument to fund training of early-stage researchers via research training programmes organised by consortia of research-performing organisations. This scheme represented about 40% of the total MCA budget, and has been the most competitive in terms of success rate (the ratio of funded projects vs. evaluated proposals). The ‘Industry-Academia Partnerships and Pathways’ (IAPP) was dedicated to the transfer of knowledge between the public and commercial/private research sectors. The ‘Individual Fellowships’ offered grants to support internationally mobile individual experienced researchers (typically post-doctoral researchers), and comprised 3 different schemes, of which the Intra-European Fellowships (IEF) addressed mobility within Europe. More detailed information on the scope and objectives of each of these schemes is available in the legal documents setting up FP7 and the ‘People’ Specific Programme [22]. The MCA are particularly appreciated by the research community for the excellent research training they offer, as well as attractive working conditions, career development and perspectives, and knowledge-exchange opportunities, at all stages of research careers, via cross-border and cross-sector mobility. Despite the diversity of schemes and target audiences, the MCA share some common features across the programme. One of them, of fundamental importance, is the centralized process of submission and evaluation of proposals against a pre-defined set of criteria, managed since 2009 by the Research Executive Agency (REA), and leading every year to the award of hundreds of grants and fellowships selected for funding. The number of MCA applications has increased dramatically in recent years, especially for the ‘Individual Fellowships’–by far the most popular actions in terms of number of submitted proposals. From 2007 to 2013, the number of ‘Individual Fellowships’ proposals submitted annually increased three-fold, from about 2600 to more than 8100 proposals. During this 7 year period, about 51,000 MCA proposals were submitted in response to more than 60 calls for proposals, accounting for almost a third of all FP7 submitted proposals (about 160,000 proposals have been submitted in the context of nearly 500 calls for proposals).

The MCA submission and evaluation process consists of the following steps: i) preparation, drafting and submission of a complete proposal by the applicant(s) before the deadline stipulated in the official documents of the calls for proposals; ii) Eligibility checks performed by the REA services, in order to ensure that the proposals submitted comply with the requirements specified in the Work Programme for the call in question; iii) Allocation of each proposal to a set of (at least) 3 external experts reviewers—raters—based on the best possible match between the expertise of available raters and the scientific field of the proposal (and also ensuring a fair representation of raters’ nationalities and gender balance), and checking conflicts of interest; iv) A remote evaluation phase, where raters assess the proposals allocated to them, against 4 (for ITN and IAPP) or 5 (for IEF) evaluation criteria, and draft an Individual Evaluation Report (IER) for each proposal, so that each proposal has (a minimum of) 3 IER; v) Consensus meetings organised in Brussels, in the presence of all raters. Each proposal is discussed by the 3 raters who had evaluated it remotely. One of the 3 raters, acting as a rapporteur, prepares a Consensus Report (CR), with the comments and scores commonly agreed by all 3 raters; vi) The creation of the Evaluation Summary Report (ESR), which is the final version of the CR sent to applicants. The ESR score always corresponds to the CR score.

The aim of the present study was to use the large set of data available from the MCA grants applications to examine its peer-review evaluation process, in particular the agreement among raters in the different phases of the evaluation workflow.

## Methods

### Data sources

The data for this study consisted of n = 24,897 proposals (n = 74,691 individual evaluation reports—reviews), representing nearly half of all applications submitted to MCA calls for proposals in FP7. The actions considered for this study were: IEF, as a representative of the ‘individual fellowships’; ITN, as a representative of large multi-beneficiary grants, and; IAPP, as a representative of schemes with a smaller number of applications. The calls selected were the following: IAPP, from 2007 to 2009 and for 2011 (4 calls); ITN, 2008 and from 2010 to 2012 (4 calls); IEF, from 2007 to 2013 (7 calls). No calls for proposals were organized for IAPP in 2010, and for ITN in 2009. Also, the ITN call in 2007 was organized as a 2-stage submission process and was thus not considered for this study. The calls for proposals for IAPP in 2012 and 2013 and for ITN in 2013 were organized with the support of a different information technology tool, which did not allow data extraction for the current study.

The data analysed in this study corresponds to steps iv) and v) of the MCA submission and evaluation process described in the previous section. All proposals for which IER and CR were recorded, i.e. which have been evaluated by raters, were included in this study, including those where the proposal was evaluated but withdrawn by the applicant or declared ineligible at any later stage (those cases represent less than 0.15% of the total number). Also, for those cases where a fourth rater was assigned to the proposal (less than 0.2% of all proposals), the fourth IER was disregarded.

Proposals were evaluated in different scientific panels, each with a separate pool of raters: Chemistry, Economic and Social Sciences/Humanities, Information Science/Engineering, Environment/Geosciences, Life Sciences, Mathematics, and Physics. In order to process the data, IER and CR scores given for each evaluation criterion, by each of the 3 raters, were extracted and sorted, together with the final score (given on a 0–100 point scale). Each criterion is scored using a scale from 0 (fail) to 5 (excellent). The final score is then calculated using weighting factors for each individual evaluation criteria and then multiplied by 20 to get the 0–100 score. IEF proposals were evaluated against 5 criteria: Science and Technology (S&T) Quality, Training, Researcher, Implementation, and Impact; whereas ITN and IAPP were evaluated against 4 criteria: S&T Quality, Training (ITN)/Transfer of Knowledge (IAPP), Implementation, and Impact. This means that, in an example of an ITN call, if a proposal gets the score of 4.2 (out of max. 5) for criterion 1, which weighs 30%, 4.7 for criterion 2 (20% weight), 3.8 for criterion 3 (30% weight) and 4.4 for criterion 4 (20% weight), then the composite score is calculated as 4.2×0.3+4.7×0.2+3.8×0.3+4.4×0.2 = 4.22; the final score is then 4.22×20 = 84.40 (out of max. 100).

### Data analysis

The final dataset was complete and did not have missing values in for individual evaluation scores. To evaluate the inter-rater agreement of the three raters who evaluated an individual proposal, we calculated the Average Deviation (AD) index [23] for each proposal. Although interpreted as a measure of inter-rater agreement [24], the AD index is actually a measure of disagreement that involves determining the average difference between scores of individual raters and the average scores of all raters. If an AD index is equal to zero, there is a perfect agreement between the raters. We decided to use this as a main measure of inter-rater agreement rather than other measures, such as intraclass correlation (ICC) or within group agreement index (r_wg_) which are sometimes used for similar situations [13,25], because AD index does not require the specification of null distribution and estimates inter-rater disagreement in the units of the original scale, making it easier to understand and interpret, and is therefore considered a more pragmatic measure [23,24]. Moreover, simulation research has singled out AD index as performing well relative to other inter-rater agreement indices [24–26]. We also calculated the intraclass correlation coefficients (ICC) which are often used in assessing raters’ agreement [13]; we used one-way random ICC because different raters rated different proposals.

Categorical data were presented as absolute and relative frequencies, and continuous data as means and standard deviations (for normally distributed data) or medians and interquartile ranges (for non-normally distributed data). Although we analysed the data for the whole population of proposals and did not perform sampling, where statistical analysis may be redundant [27], we compared the datasets from different evaluation panels and calls. One-way ANOVA was used to test differences in CR scores between panels, paired samples t-test was used to test differences between the CR and the average of the 3 Individual Evaluation Reports (AVIER) scores. Pearson's correlation coefficient was used to test bivariate associations (between CR and AVIER, inter-correlations of IER criteria of different raters and between IER and CR scores for separate criteria) and principal component analysis was used to investigate the latent structure of IER criteria ratings. Simple linear regression analyses were used to assess the relationship of CR or AVIER as a dependent and AD index as a predictor variable. Because of the size of the dataset, the level of significance was set at p<0.01 for all statistical tests. All statistical analyses were performed using SPSS 17 for Windows (SPSS Inc., Chicago, IL, USA).

## Results

Overall, the mean (± standard deviation, SD) final CR scores for all proposals was 79.8 ±11.0 (0–100 scale), showing a bell shaped distribution skewed to the left, i.e. towards higher scores (Fig A in S1 File), with consistently lower scores in Economic and Social Sciences/Humanities, Information Science/Engineering and Mathematics panels (Table 1). There were no significant differences in CR or AVIER scores over different years (Fig B in S1 File.).

**Table 1:** Mean consensus report (CR) scores (±standard deviation, SD) across evaluation panels for all proposals and for proposals with disagreements among raters in their Individual Evaluation Report (IER) score and between Consensus Report (CR) and average Individual Evaluation Report (AVIER) scores.

Panel	Mean score (±SD) in proposals where:				
	Total*	All raters agree	One rater differs^§^	All raters differ^¶^	Mean score (±SD) in proposals with AVIER vs CR difference**
**Total**	**79.8 ±11.0 (n = 24897)**	**81.0±10.1 (n = 21398)**	**74.0±13.1 (n = 1424)**	**70.9±12.8 (n = 2075)**	**69.3±19.8 (n = 368)**
Chemisty	81.0±9.8 (n = 2665)	81.9±9.2 (n = 2362)	75.3±13.2 (n = 132)	73.2±10.0 (n = 171)	70.6±19.9 (n = 32)
Economic and Social Sciences/Humanities	78.1±12.9† (n = 4677)	79.8±12.4 (n = 3646)	74.6±13.1 (n = 431)	70.7±12.9 (n = 600)	73.1±19.5 (n = 142)
Information Science/Engineering	76.9±11.9‡ (n = 2983)	78.3±11.1 (n = 2478)	70.9±13.7 (n = 199)	69.2±12.7 (n = 306)	62.7±18.0 (n = 50)
Environment	80.4±10.4 (n = 3243)	81.5±9.4 (n = 2860)	74.5±13.3 (n = 153)	70.1±13.8 (n = 230)	66.1±20.9 (n = 42)
Life Sciences	80.9±10.3 (n = 7658)	82.0±9.4 (n = 6785)	74.5±13.3 (n = 354)	71.4±13.2 (n = 519)	65.8±20.4 (n = 71)
Mathematics	78.2±10.2† (n = 731)	79.6±8.6 (n = 623)	71.1±15.2 (n = 41)	69.2±13.6 (n = 67)	79.1±9.6 (n = 5)
Physics	80.8±9.2 (n = 2940)	81.6±8. (n = 2644)	75.3±11.7 (n = 114)	72.4±12.0 (n = 182)	72.4±17.9 (n = 26)

*Mean consensus report scores were compared only for all proposals (One-way ANOVA: F_6,24890_ = 81.5, p<0.001)

^†^Significantly lower than Chemistry, Environment, Life Sciences and Physics panels (Tukey post-hoc test, p<0.001 for all comparisons).

^‡^Significantly lower than Chemistry, Economic and Social Sciences/Humanities, Environment, Life Sciences and Physics panels (Tukey-post hoc test, p<0.001 for all comparisons).

^§^Disagreement is defined as one rater differing 10 or more points from other two raters, who agree within 5 points (scale 0–100).

^¶^Disagreement is defined as all raters differing 10 or more points (scale 0–100).

**Disagreement is defined equal or greater than 10 points (scale 0–100).

We first tested the association between the CR and AVIER scores. Pearson's correlation coefficients for all comparisons were around 0.95 and above, indicating a nearly perfect correlation between the CR and AVIER scores. (Table 2). We next examined whether there were any systematic differences between the CR and AVIER scores.

**Table 2:** Associations and differences between consensus reports (CR) and average individual evaluation report (AVIER) scores and inter-rater agreement (average deviation index, AD index) for all proposals, and for different actions and panels*.

Actions and panels (No. proposals)*	r_CR/AVIER_ †	Difference CR-AVIER‡ (p§)	Median AD index (interquartile Q1–Q3 range)
**All proposals (n = 24897)**	0.957	0.30 (<0.001)	5.4 (3.4–8.3)
IAPP (n = 759)	0.970	-0.58 (<0.001)	7.3 (4.6–10.6)
IEF (n = 20593)	0.958	0.38 (<0.001)	5.2 (3.3–8.0)
ITN (n = 3545)	0.946	0.07 (0.319)	6.3 (3.9–9.6)
**IAPP:**			
Chemistry (n = 63)	0.974	-0.89 (0.053)	7.2 (3.9–11.0)
Economic and Social Sciences/Humanities (n = 68)	0.966	-0.61 (0.284)	7.2 (4.7–11.3)
Information Science/Engineering (n = 296)	0.966	-0.29 (0.154)	7.3 (4.3–10.3)
Environment/Geosciences (n = 84)	0.972	-1.37 (0.003)	7.6 (4.6–10.9)
Life Sciences (n = 203)	0.973	-0.72 (0.009)	7.8 (5.6–10.9)
Mathematics (n = 6)	0.994	-0.68 (0.594)	8.8 (5.3–13.4)
Physics (n = 39)	0.974	0.21 (0.617)	7.0 (4.0–9.8)
**IEF:**			
Chemistry (n = 2204)	0.963	0.51 (<0.001)	4.9 (3.1–7.3)
Economic and Social Sciences/Humanities (n = 4228)	0.945	0.71 (<0.001)	6.7 (4.2–10.0)
Information Science/Engineering (n = 1888)	0.962	-0.00 (0.967)	5.7 (3.5–8.4)
Environment/Geosciences (n = 2731)	0.955	0.41 (<0.001)	4.9 (3.1–7.5)
Life Sciences (n = 6408)	0.964	0.22 (<0.001)	4.8 (3.0–7.3)
Mathematics (n = 665)	0.966	0.20 (0.054)	5.3 (3.2–8.2)
Physics (n = 2469)	0.966	0.39 (<0.001)	4.7 (2.9–7.1)
**ITN:**			
Chemistry (n = 398)	0.909	0.11 (0.611)	6.2 (3.7–9.6)
Economic and Social Sciences/Humanities (n = 381)	0.940	-0.43 (0.094)	7.6 (5.0–11.0)
Information Science/Engineering (n = 799)	0.953	0.12 (0.360)	6.7 (4.5–9.6)
Environment/Geosciences (n = 428)	0.950	0.15 (0.400)	5.8 (3.4–9.3)
Life Sciences (n = 1047)	0.953	0.14 (0.228)	6.3 (3.8–9.4)
Mathematics (n = 60)	0.903	0.67 (0.142)	7.6 (4.5–11.3)
Physics (n = 432)	0.925	0.02 (0.921)	5.5 (3.4–8.8)

*Abbreviations: IAPP—Industry-Academia Partnerships and Pathways, IEF—Intra-European Fellowships, ITN—Initial Training Networks.

^†^p<0.001 for all (Pearson correlation).

^‡^Difference between the scores on the scale from 0–100.

^§^Paired samples t-test.

Differences were negligible (about 1 point on 0–100 scale), varied non-systematically in both directions (Table 2), and showed a normal distribution (Fig C in S1 File). Furthermore, 61.4% of all proposals had less than 2 points difference between AVIER and CR scores. The fact that they reached statistical significance was a result of the very high number of proposals and did not reveal any practically meaningful differences between the CR and AVIER scores. The AD index for individual evaluation criteria was also very low, indicating that raters agreed very well in their independent evaluations of the proposals (Table A in S1 File).

**Fig 1:**
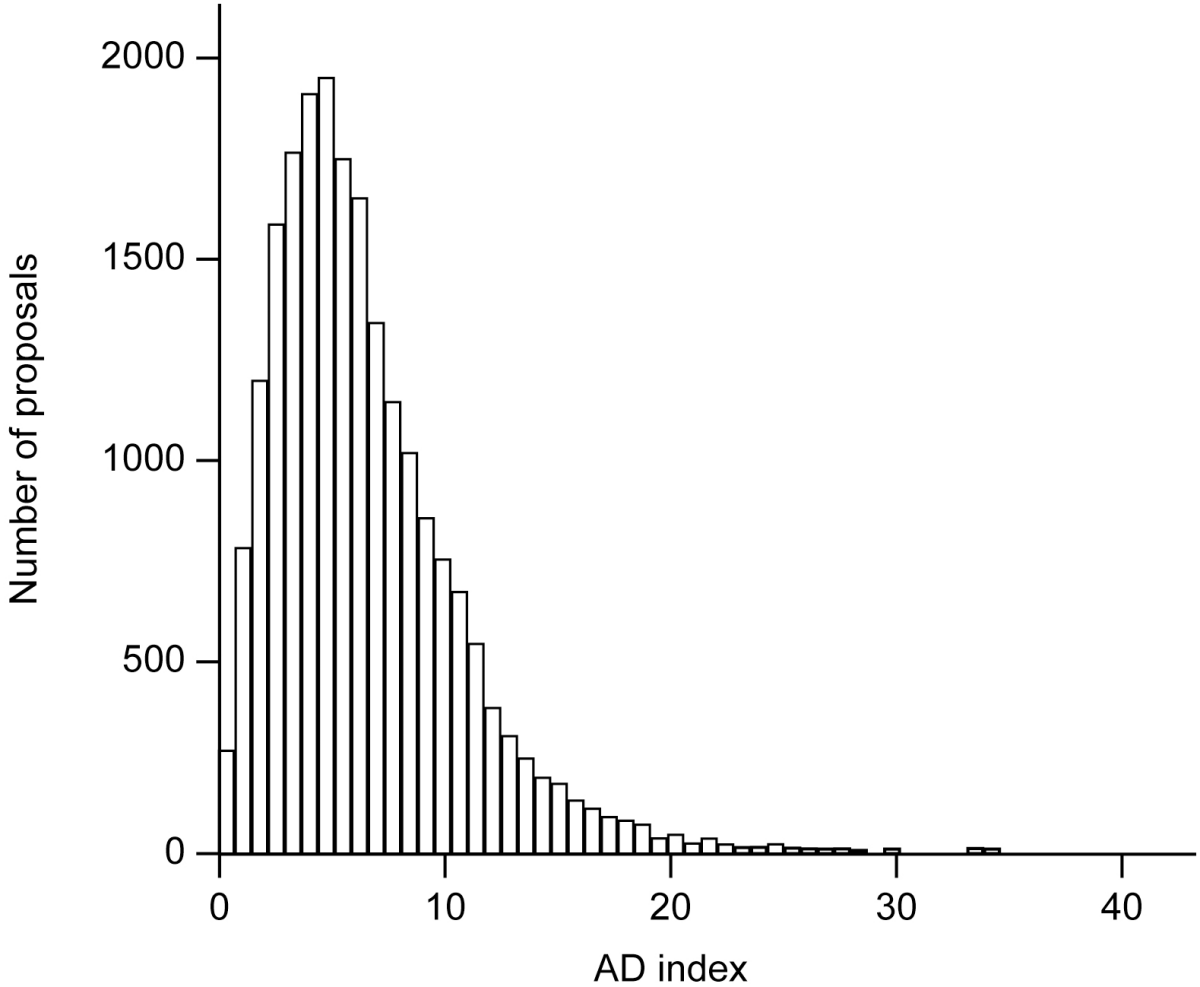
Distribution of average deviation (AD) indices for all proposals. Positively skewed distribution shows that the majority of proposals had AD index below 10 points (20,988 out of 24,897 proposals or 84.3%).

We also calculated the AD index for the IER scores. Distribution of AD indices was positively skewed for all proposals, as well as for all subgroups of proposals, showing very good inter-rater agreement for the majority of proposals (Fig 1). Medians and interquartile ranges were therefore used as descriptives (Table 2). Median AD indices showed good general agreement among raters. The overall median AD index was 5.4 points (on a scale 0–100), and for three quarters of all proposals it was equal or below 8.3 points. We also calculated one-way random intraclass correlation coefficients for individual evaluators’ reports (IER) for all proposals (ICC = 0.67, 95%CI = 0.66–0.68) and for separate evaluation panels. Consistently with AD indices, ICCs indicated good inter-rater agreement (Table A in S1 File).

There were, however, cases where the agreement was lower. Possible disagreements could be due to two typical situations—either one rater scores a proposal in a completely different way than the other two raters, or all three raters differ in their scores. To test these possibilities, we first isolated the proposals where there was high agreement between two out of three raters. We defined the cut-off for this difference in the following way: 1) two raters agreed if the difference between their scores was less than or equal to 5 –because 5.4 was the median AD for all proposals; and 2) the third rater disagreed with other two if the AD index for the proposal was equal to or greater than 10 –because this would put it above the third quartile of all the AD indices for IER scores. There were 1424 (5.7%) cases (Table 3). Pearson’s correlation between CR and AVIER scores, although somewhat lower than for the total population of proposals, was still very high (r_CR/AVIER_ = 0.913, p<0.001). The difference between CR and AVIER scores, although statistically significant due to large sample size, was again extremely small (CR—AVIER = 1.1 on a 0–100 scale; p<0.001, paired samples t-test). The findings were similar across evaluation panels and calls (Table B in S1 File).

**Table 3:** Distribution of proposals with disagreements among raters in their Individual Evaluation Report (IER) score and between Consensus Report (CR) and average Individual Evaluation Report (AVIER) scores across evaluation panels*

	No. proposals (row %) with disagreement		
Panel (No. proposals)	One rater differs*	All raters differ†	Difference in AVIER vs CR‡
Chemistry (n = 2665)	132 (5.0)	171 (6.4)	32 (1.2)
Economic and Social Sciences/Humanities (n = 4677)	431 (9.2)	600 (12.8)	142 (3.0)
Information Science/Engineering (n = 2983)	199 (6.7)	306 (10.3)	50 (1.7)
Environment/Geosciences (n = 3243)	153 (4.7)	230 (7.1)	42 (1.3)
Life Sciences (n = 7658)	354 (4.6)	519 (6.8)	71 (0.9)
Mathematics (n = 731)	41 (5.6)	67 (9.2)	5 (0.7)
Physics (n = 2940)	114 (3.9)	182 (6.2)	26 (0.9)
**Total (n = 24897)**	**1424 (5.7)**	**2075 (8.3)**	**368 (1.5)**

*Disagreement is defined as one rater differing 10 or more points from other two raters, who agree within 5 points (scale 0–100).

^†^Disagreement is defined as all raters differing 10 or more points (scale 0–100).

^‡^Disagreement is defined equal or greater than 10 points (scale 0–100).

Next we isolated the proposals where there was a disagreement between all three raters, defined as the difference between each pair of IER scores equal to or greater than 10 points, putting them above the third quartile of all AD indices. There were 2075 (8.3%) cases (Table 3). Pearson’s correlation between CR and AVIER scores was again very high (r_CR/AVIER_ = 0.917, p<0.001). The difference between CR and AVIER scores was not statistically significant (CR-AVIER = 0.2; p = 0.093, paired samples t-test). The findings were similar across evaluation panels, but IAPP and ITN had a consistently a greater percentage of proposals with differences among raters when compared to IEF (Table B in S1 File).

**Fig 2:**
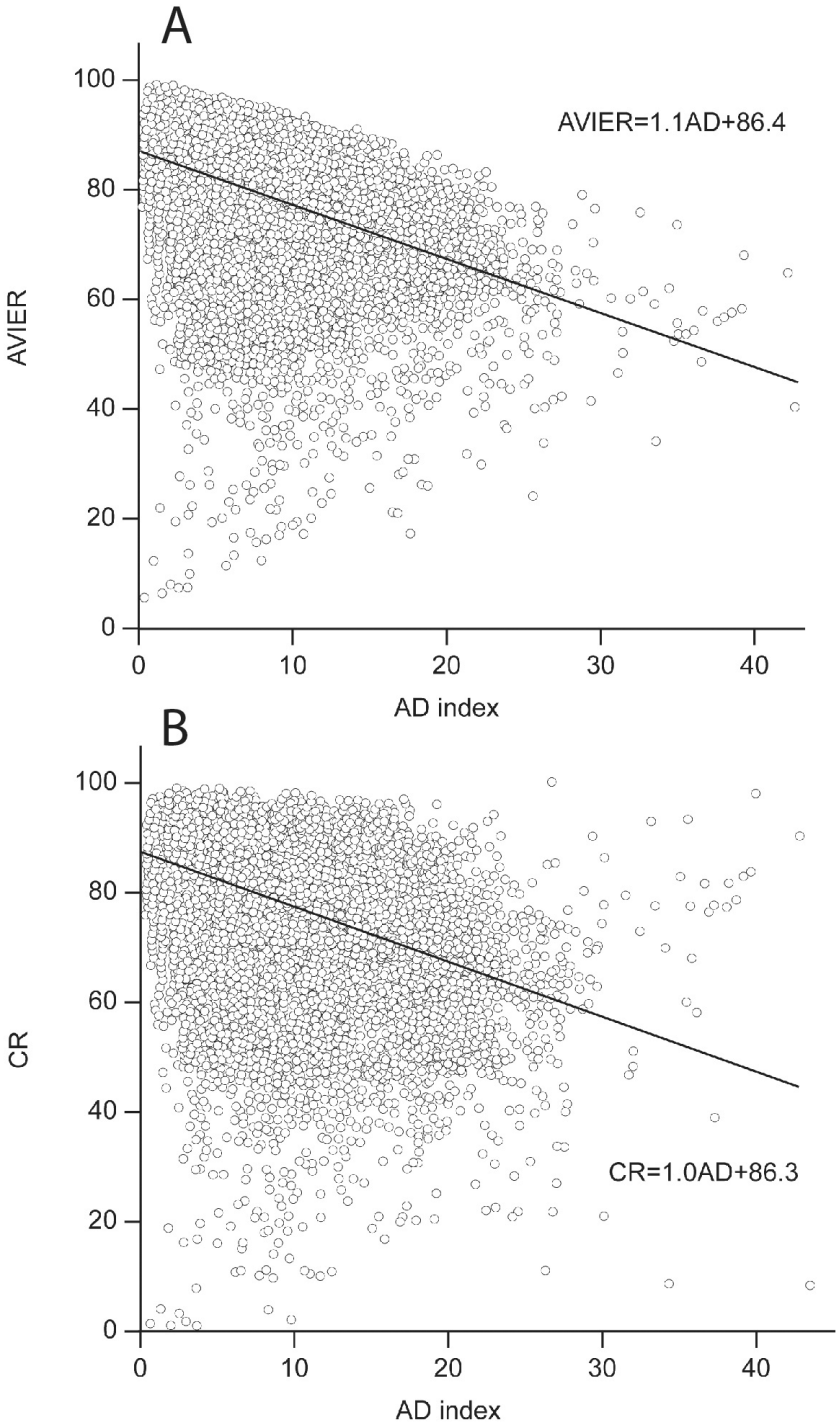
Association between raters' agreement (AD index—lower score means greater agreement) and the average Individual Evaluation Report (AVIER) (A) or consensus report (CR) (B). Line indicates the regression line. Circles—individual proposals.

To assess the relationship between AD indices as a measure of inter-rater agreement and the scores of the proposals, we first performed a simple linear regression analysis with CR or AVIER scores as a dependent and AD index as a predictor variable. Both measures were significantly and negatively associated with AD index (AVIER = 86.4–1.1×AD, R^2^ = 0.19, p<0.001 and CR = 86.3–1.0×AD, R^2^ = 0.15, p<0.001; Fig 2), indicating more disagreement for proposals with lower scores. We found statistically significant differences in CR scores depending on the initial agreement (Table 1) and grouped together the proposals where either one rater differed from the other two who agreed or where all of them differed among themselves and compared this subgroup to all other proposals. CR scores were on average 9 points lower in cases where raters initially disagreed (no initial disagreement: M±SD = 81.0±10.1 vs. initial disagreement: M±SD = 72.3±13.0; p<0.001, independent samples t-test).

We also isolated a group of proposals where the absolute difference between the CR and the AVIER scores was equal to or greater than 10 points (Table 3). There were 368 (1.5%) cases. Positive and negative differences were equally distributed (180 or 48.9% positive and 188 or 51.1% negative differences), indicating that, when the CR corrected the initial AVIER score, it was as likely to increase it as to decrease it. However, these proposals had significantly lower CR scores than other proposals (69.3±19.8 vs 79.8±11.0, respectively; p<0.001, independent samples t-test). The findings were similar across scientific panels but again IAPP and ITN calls had more proposals with such differences when compared to IEF (Table B in S1 File). For all subgroups’ comparisons (Table 2, Table B in S1 File), the Economic and Social Sciences/Humanities panel had a higher proportion of proposals with disagreements among raters and differences between the CR and AVIER scores.

To investigate whether there was a possible pattern in scoring individual IER criteria (5 for IEF and 4 for ITN or IAPP), we investigated the inter-correlations of these criteria (Table 4 and Table C in S1 File). Our interest was in comparing the correlation of the scores of different raters for the same criterion and correlations of the same rater's scores of different criteria across proposals. If correlations of different raters’ scores for the same criterion were high it would mean that, by those criteria, they assess specific characteristics of the proposal with a similar level of objectivity (i.e. different raters score the same item similarly). If, on the other hand, correlations of the same rater’s scores of different criteria were high, it would imply that a rater decides upon a general score for the proposal and uses that score as a reference point to score each individual criterion. We found low correlations between different rater's scores for the same criterion and the same proposal and high correlations of the same rater's scores of different criteria for the same proposal (Table 4), which indeed suggests that raters scored proposals in a more holistic way and, generally, assessed each criterion in relation to the other criteria for the same proposal (Table C in S1 File).

**Table 4:** Pearson’s inter-correlations of IER criteria of different raters*.

	Rater 1					Rater2					Rater 3				
	S&T quality	Training/ToK	Researcher	Implementation	Impact	S&T quality	Training/ToK	Researcher	Implementation	Impact	S&T quality	Training/ToK	Researcher	Implementation	Impact
**Rater 1:**	1	0.698	0.600	0.668	0.693	0.291	0.279	0.231	0.278	0.274	0.296	0.290	0.231	0.289	0.282
**S&T quality**															
**Training/Transfer of Knowledge**		1	0.582	0.718	0.740	0.282	0.361	0.248	0.319	0.324	0.270	0.357	0.236	0.324	0.320
**Researcher**			1	0.582	0.646	0.217	0.231	0.293	0.230	0.241	0.234	0.246	0.306	0.249	0.251
**Implementation**				1	0.740	0.281	0.330	0.247	0.360	0.328	0.282	0.335	0.254	0.367	0.330
**Impact**					1	0.278	0.325	0.251	0.318	0.341	0.277	0.327	0.260	0.328	0.341
**Rater 2:**															
**S&T quality**						1	0.694	0.590	0.668	0.685	0.295	0.286	0.230	0.285	0.276
**Training/Transfer of Knowledge**							1	0.583	0.713	0.734	0.287	0.369	0.250	0.335	0.328
**Researcher**								1	0.564	0.639	0.228	0.240	0.294	0.244	0.244
**Implementation**									1	0.730	0.282	0.332	0.245	0.367	0.330
**Impact**										1	0.275	0.322	0.256	0.329	0.342
**Rater 3:**															
**S&T quality**											1	0.695	0.606	0.665	0.690
**Training/Transfer of Knowledge**												1	0.589	0.710	0.737
**Researcher**													1	0.573	0.645
**Implementation**														1	0.733
**Impact**															1

*Evaluation criteria: Science and Technology (S&T) quality; Training (for ITN and IEF) or Transfer of Knowledge (ToK, for IAPP); Researcher (criterion used only for IEF); Implementation; Impact. N = 24897 for all except for “Researcher” where n = 20593. All correlations were statistically significant at p<0.001 level.

Finally, to take this analysis a step further, we performed a principal components analysis with the evaluation criteria. This procedure is usually done when investigating a latent structure that underlies a set of items (in our case, criteria scored by three raters). The analysis is performed on a set of scores of different items and the resulting solution can reveal a smaller number of components, or factors, that underlie individual scores. In other words, resulting components may be understood as an empirical suggestion as to how the items can be grouped together based on what they actually measure. In our case, if criteria represented measures of specific, i.e. different, aspects of proposals that were objectively measured, we would expect four (ITN and IAPP calls)/five (IEF call) components corresponding to the four/five criteria, with each component including the three raters’ scores of the same characteristic or aspect (i.e. criterion) of proposals.

The results from the analysis of the data pointed in another direction, already visible in the inter-correlation matrices (Table 4, S3 Table). We extracted three components, each representing a single rater, which confirmed our previous conclusion that criteria scores reflected the rater’s global score rather than specific aspects of the proposal. The three-component solution explained large portion of variance (73%) and component loadings were very high (all above 0.7).

## Discussion

Our analysis of a large dataset of grant proposals submitted to the Marie Curie Actions over the FP7 lifetime demonstrates a good internal consistency and overall high agreement among expert reviewers—raters. It is difficult to compare our results directly to other studies that analysed reliability of individual vs panel grant reviews, mainly because of the differences in the review processes, the number of raters reviewing a single proposal and statistical approaches [8,14]. Furthermore, the MCA evaluation procedure does not include a common panel assessing more than one proposal, but rather operates with groups of raters (almost always composed of 3 experts) discussing their evaluation—consensus meetings—for each individual proposal. Studies that looked at the value of a larger panel [8,14] showed that panel discussions of proposals contribute to the reduction of disagreement among individual raters but do not contribute to the overall improvement of reliability of the final score. In our study, the agreement between raters was in general very high, both among their independent assessments and when these were compared to the score reached after the consensus meetings’ discussion during the central meeting. This finding was consistent over different types of grant schemes and over the years of the MCA. It may at least in part be related to the high quality of the submitted proposal and high competitiveness of MCA calls, as indicated by generally high scores for the whole study population (distribution of scores shifted to high scores, S1 Fig). Overall, disagreement was greater for proposals with lower scores. Also, when raters initially disagreed in their independent evaluation, this disagreement influenced the final scores, which were generally lower. Our finding on greater disagreement of raters for proposals with lower scores contrasts the study of Cicchetti [17], who reported greater agreement of reviewers for grants with lower ratings, as well as for rejection recommendation in scientific journals. The differences may be related to distinct peer review processes in grant programmes and in journals. For example, greater reviewer’s agreement on manuscript rejection in Ciccheti study concerned predominantly psychology journals [17], whereas medical journals also report either higher agreement for acceptance than rejection recommendation (84% vs. 31% agreement rate in a general medical journal [28]) or poor agreement on any recommendation [29].

Intraclass correlation coefficient for IERs and individual evaluation criteria were also high, confirming high agreement among rates and complementing the data from AD index analysis. More recently, comparison of average deviation measures against theoretically expected distributions were suggested [24]. We could not directly compare AD indices for IER from our study to published significance criteria for different null distributions because IER scores are on a 0–100 scale and the published theoretical criteria provide cut-off values for scales with 4, 5 or 7 categories. When we transformed IER scores to a 5 point scale and calculated AD indices, median AD index was 0.27 (Q1–Q3 = 0.16–0.41, which was lower than cut-off values for all published null-distributions [24], indicating statistically significant agreement at p<0.05 level.

The consistency and high agreement of raters’ scores, as well as the consistency of an individual rater’s scores across individual evaluation criteria, indicate that, at least for some of the proposals, the remote assessments and its average score (AVIER) can provide reliable final judgment of the proposal. This is an important finding in view of the high competition for MCA funding, particularly for the individual fellowships scheme. In times of financial crisis, national governments rarely increase their budget for research and innovation. Therefore, Horizon 2020, the new EU Framework Programme for Research and Innovation for the period 2014–2020, is perceived by many as the main instrument to fund competitive research at the European level. As a consequence of that, Horizon 2020 funding schemes may face oversubscription, with a possible increase in the number of applications for grants over the years. This means that the current evaluation process will struggle to be both financially and logistically sustainable, as more proposals submitted imply more experts, more time-consuming review processes, and more constraints from the logistical point of view (e.g. space and facilities to organize the consensus meetings). Previous studies support the rationale of using more remote, distance-based, approaches as a good alternative for peer review processes, given the environmental impact and costs savings involved when compared to on-site evaluations [30]. The results of our study demonstrate that it may be safe and appropriate, across the different types of schemes and evaluation panels, to predominantly use a remote-based evaluation process and the average score of individual assessments as the final proposal’s score. This is especially true for the individual-driven grant applications. Our study also identifies sets of proposals, which constitute about 15% of the proposals’ population that may need more discussion in order to reach consensus on the final score. These proposals are identified as those which have a large difference between one vs. the other two raters, large differences among all three raters, or those with a large difference between the average of the initial (remote) scores (AVIER) and the final consensus score. Furthermore, IAPP and ITN calls had a greater number of proposals with disagreements, demonstrating that the evaluation of complex proposals, involving partnerships of several research groups with multidisciplinary and inter-sectorial features, require a more elaborate review procedure. As the number of proposals submitted to ITN and IAPP is much smaller than to the individual fellowships, a two-step review procedure, as presently in place, with both remote and consensus phases, appears to be justified for these schemes. Consensus meetings and discussion among raters may be especially relevant for the evaluation of panels in the field of Economic Sciences, Social Sciences and Humanities due to the significantly higher proportion of proposals with disagreement. Future research should be directed to more qualitative approaches to understanding reviewers’ decision-making processes and their reasoning during consensus meetings, as well as differences among different research fields. Rare qualitative studies into grant application peer review process have uncovered important aspects of peer review as a social phenomenon involving collaborative decision-making processes, social expectations and professional interests and interpersonal issues [7,11].

Our study was retrospective and we cannot make claims on what influences scoring or agreement among reviewers. Also, AD index has a limitation as a measure of inter-rater reliability because it represents disagreement in units of a rating scale. Therefore the AD indices obtained in our study may not be comparable to other studies where raters used different scales. Although there are suggestions for more refined approaches to estimating inter-rater reliability in cases like our study [31,32], we decided to use the AD index because of its simplicity and straightforwardness in interpretation [23–26]. Despite these limitations, we can be confident in our observations and on the reliability of our results because of the very large sample of data accumulated over 7 years.

In conclusion, this study provides the first comprehensive analysis of the grant review process of one of the major EU research granting programmes. It demonstrates the consistency and reliability of the MCA review process and identifies critical parts that can be simplified and improved without impacting on the high quality and reliability of its outcome. The question remains whether the analysis of the peer review procedure can be used to predict outcome measure of research success, such as publications, citations and/or patents [33]. We recommend continuous quality assurance analyses, such as the one performed in this study, as useful tools to follow and analyse trends of peer review processes and their relation to research outcomes in order to allow adequate and more efficient decisions on evaluation procedures to ensure excellence in research.

## Supporting Information

S1 File Description of proposals and inter-reater agreements. Fig A: Distribution of proposals by their Consensus Report (CR) scores. Fig B: Mean Consensus Reports (CR) scores for different years. Fig C: Distribution of proposals by their differences between Consensus Reports (CR) and average Individual Evaluation Reports (AVIER) scores. Table A. Inter-rater agreement (average deviation index, AD index) for individual evaluation criteria across all evaluation panels. Table B. Distribution of proposals, across panels and type of action, where a) one rater disagrees with other two raters; b) all raters disagree with each other; c) difference between the Consensus Report (CR) and average Individual Evaluation Report (AVIER) score is large. Table C. Pearson’s correlations between IER and CR scores for separate criteria.
(DOC)

## References

[1] Weller AC. Editorial peer review: Its strengths and weaknesses. Medford, NJ: Information Today Inc.; 2001.

[2] Jefferson T, Rudin M, Brodney Folse S, Davidoff F. Editorial peer review for improving the quality of reports of biomedical studies. Cochrane Database Syst Rev. 2007;2: MR000016 17443635 10.1002/14651858.MR000016.pub3 PMC8973931

[3] Demicheli V, Di Pietrantonj C. Peer review for improving the quality of grant applications. Cochrane Database Syst Rev. 2007;2: MR000003 17443627 10.1002/14651858.MR000003.pub2 PMC8973940

[4] Malički M, von Elm E, Marušić A. Study design, publication outcome, and funding of research presented at international congresses on peer review and biomedical publication. JAMA. 2014;311: 1065–1067. 10.1001/jama.2014.143 24618970

[5] Wessely S (1998) Peer review of grant applications: What do we know? Lancet. 1998;352: 301–305. 9690424 10.1016/S0140-6736(97)11129-1

[6] Marsh HW, Jayasinghe UW, Bond NW. Improving the peer-review process for grant applications. Am Psychol. 2008;63: 160–168. 10.1037/0003-066X.63.3.160 18377106

[7] Abdoul H, Perrey C, Amiel P, Tubach F, Gottot S, Durand-Zaleski I, et al Peer review of grant applications: Criteria used and qualitative study of reviewer practices. PLoS ONE. 2012;7: e46054 10.1371/journal.pone.0046054 23029386 PMC3460995

[8] Fogelholm M, Leppinen S, Auvinen A, Raitanen J, Nuutinen A, Väänänen K. Panel discussion does not improve reliability of peer review for medical research grant proposals. J Clin Epidemiol. 2012;65: 47–52. 10.1016/j.jclinepi.2011.05.001 21831594

[9] Graves N, Barnett AG, Clarke P. Funding grant proposals for scientific research: retrospective analysis of scores by members of grant review panel. BMJ. 2011;343: d4797 10.1136/bmj.d4797 21951756 PMC3181233

[10] Johnson VE. Statistical analysis of the National Institutes of Health peer review system. Proc Nat Acad Sci USA. 2008;105: 11076–11080. 10.1073/pnas.0804538105 18663221 PMC2488382

[11] Kaatz A, Magua W, Zimmerman DR, Carnes M. A quantitative linguistic analysis of National Institutes of Health R01 application critiques from investigators at one institution. Acad Med. 2014, e-pub ahead of print. 10.1097/ACM.0000000000000442 PMC4280285 25140529

[12] Mayo NE, Brophy, Goldberg MS, Klein MB, Miller S, Platt RW, Ritchie J. Peering at peer review revealed high degree of chance associated with funding of grant applications. J Clin Epidemiol. 2006;59: 842–848. 16828678 10.1016/j.jclinepi.2005.12.007

[13] Mutz R, Bornmann L, Daniel H-D. Heterogeneity of inter-rater reliabilities of grant peer reviews and its determinants: A general estimating equations approach. PLoS ONE. 2012;7: e48509 10.1371/journal.pone.0048509 23119041 PMC3485362

[14] Olbrecht M, Tibelius K, D’Aloisio. Examining the value added by committee discussion in the review of applications for research awards. Research Evaluation. 2007;16: 79–91.

[15] Pederson T. The “study” role of past National Institutes of Health study sections. Mol Biol Cell. 2012;23: 3281–3284. 10.1091/mbc.E12-06-0448 22936698 PMC3431932

[16] Lamont M, Huutoniemi K. Comparing customary rules of fairness: evaluative practices in various types of peer review panels In: Camic C, Gross N, Lamont M, eds. Social knowledge in the making. Chicago: University of Chicago Press; 2011 pp 209–232.

[17] Cicchetti DV. The reliability of peer review for manuscript and grant submissions: a cross-disciplinary investigation. Behav Brain Sci. 1991;14: 119–135.

[18] Hodgson C. How reliable is peer review? An examination of operating grant proposals simultaneously submitted to two similar peer review systems. J Clin Epidemiol. 1997;50:1189–95. 9393374 10.1016/s0895-4356(97)00167-4

[19] Martin MR, Kopstein A, Janice JM. An analysis of preliminary and post-discussion priority scores for grant applications peer reviewed by the Center for Scientific Review at the NIH. PLoS ONE. 2010;5: e13526 10.1371/journal.pone.0013526 21103331 PMC2984433

[20] Jayasinghe UA, Marsh HW, Bond N. A multilevel cross-classified modelling approach to peer review of grant proposals: the effects of assessor and researcher attributes on assessor ratings. J Royal Stat Soc A, 2003;166:279–300.

[21] European Commission. Decision 1982/2006/EC of the European Parliament and of the Council of 18 December 2006 concerning the Seventh Framework Programme for research, technological development and demonstration activities (2007–2013). Official Journal of the European Union. 2006;L412: 1–41.

[22] European Commission. Council Decision 2006/973/EC of 19 December 2006 concerning the specific programme “People” implementing the Seventh Framework Programme of the European Community for research, technological development and demonstration activities (2007–2013). Official Journal of the European Union. 2006;L400: 270–298.

[23] Burke MJ, Finkelstein LM, Dusig MS. On average deviation indices for estimating interrater agreement. Organizational Research Methods. 1999;2: 49–68.

[24] Smith-Crowe K, Burke MJ, Kouchaki M, Signal S. Assessing interrater agreement via the average deviation index given a variety of theoretical and methodological problems. Organiz Res Meth. 2013;16: 127–51.

[25] Smith-Crowe K, Burke MJ, Cohen A, Doveh E. Statistical significance criteria for the rWG and average deviation interrater agreement indices. J Applied Psych. 2014;99: 239–61. 10.1037/a0034556 24099346

[26] Roberson QM, Sturman MC, Simons TL. Does the measure of dispersion matter in multilevel research? A comparison of the relative performance of dispersion indexes. Organiz Res Meth. 2007;10: 564–88.

[27] Cohen J. The Earth is round (p < .05). Am Psychol 1994;12: 997–1003.

[28] Baethge C, Franklin J, Mertens S. Substantial agreement of referee recommendations at a general medical journal—a peer review evaluation at Deutsches Ärzteblatt International. PLoS One. 2013;8:e61401 10.1371/journal.pone.0061401 23658692 PMC3642182

[29] Rothwell PM, Martyn CN. Reproducibility of peer review in clinical neuroscience. Is agreement between reviewers any greater than would be expected by chance alone? Brain. 2000;123:1964–9. 10960059 10.1093/brain/123.9.1964

[30] Gallo SA, Carpenter AS, Glisson SR. Teleconference versus face-to-face scientific peer review of grant application: effects on review outcomes. PLoS ONE. 2013;8: e71693 10.1371/journal.pone.0071693 23951223 PMC3740535

[31] Chao J, Stokes SL, Zhang S. A Bayesian approach to ranking and rater evaluation: an application to grant reviews. J Educ Behav Stat. 2010;35: 194–214.

[32] Giraudeau B, Leyrat C, Le Gouge A, Léger J, Caille A. Peer review of grant applications: a simple method to identify proposals with discordant reviews. PLoS ONE. 2011;6: e27557 10.1371/journal.pone.0027557 22110670 PMC3215721

[33] Li D, Agha L. Big names or big ideas: Do peer review panels select the best science proposals? Science. 2015;348:434–8. 10.1126/science.aaa0185 25908820

