# Supplementary material for: Peer Review Evaluation Process of Marie Curie Actions under EU’s Seventh Framework Programme for Research
Source: PLoS One. 2015 Jun 30;10(6):e0130753. doi: 10.1371/journal.pone.0130753 (PMC4488366; doi:10.1371/journal.pone.0130753)
Supplement: S1 File — Fig A: Distribution of proposals by their Consensus Report (CR) scores. Fig B: Mean Consensus Reports (CR) scores for different years. Fig C: Distribution of proposals by their differences between Consensus Reports (CR) and average Individual Evaluation Reports (AVIER) scores. Table A. Inter-rater agreement (average deviation index, AD index) for individual evaluation criteria across all evaluation panels. Table B. Distribution of proposals, across panels and type of action, where a) one rater disagrees with other two raters; b) all raters disagree with each other; c) difference between the Consensus Report (CR) and average Individual Evaluation Report (AVIER) score is large. Table C. Pearson’s correlations between IER and CR scores for separate criteria. (DOC) [file pone.0130753.s001.doc]

**Figure S1.** Distribution of proposals by their Consensus Report (CR) scores (N = 24897, mean = 79.8, standard deviation = 11.0).


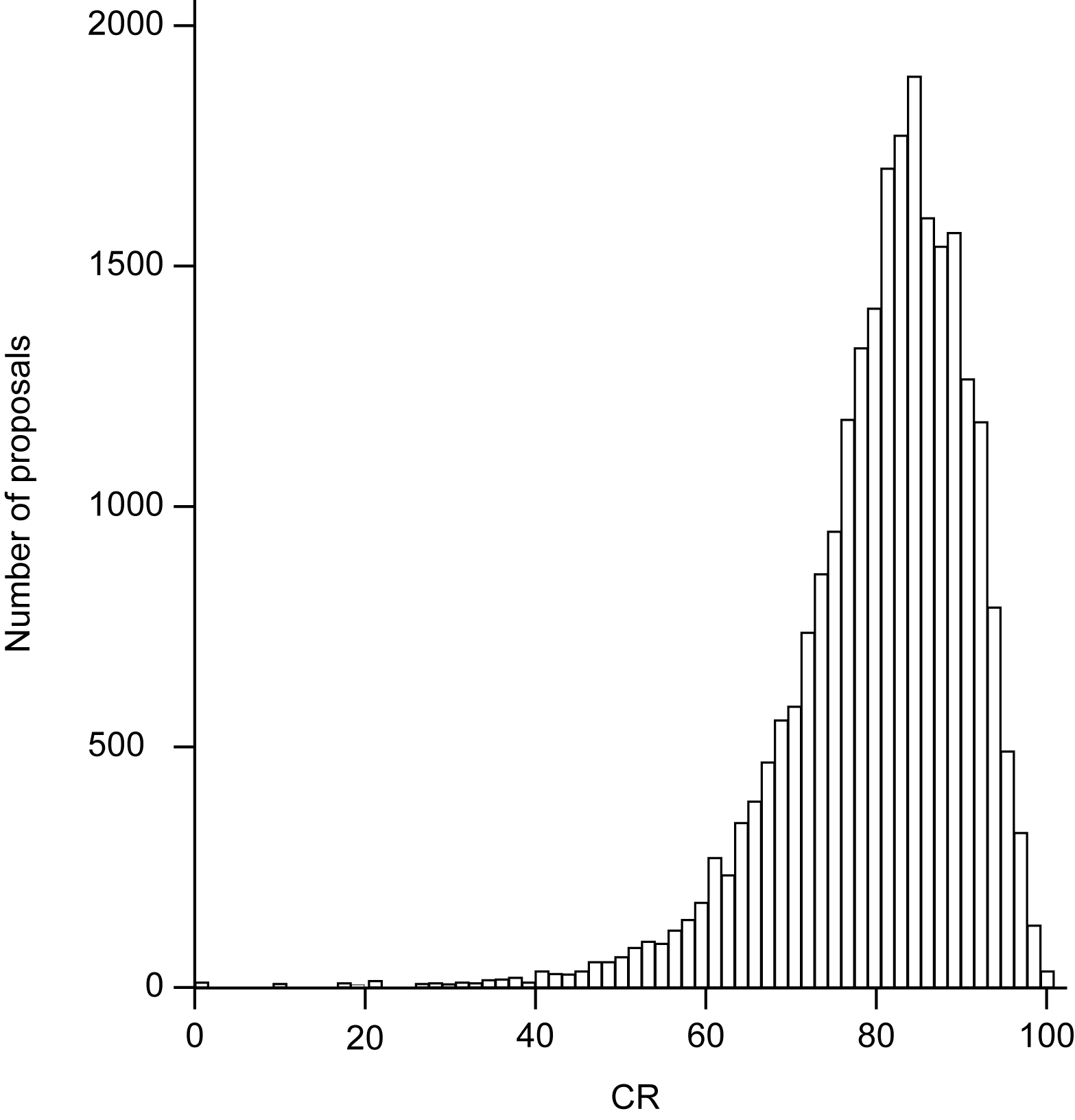


**Figure S2.** Mean Consensus Reports (CR) scores for different years (N=24897).

**
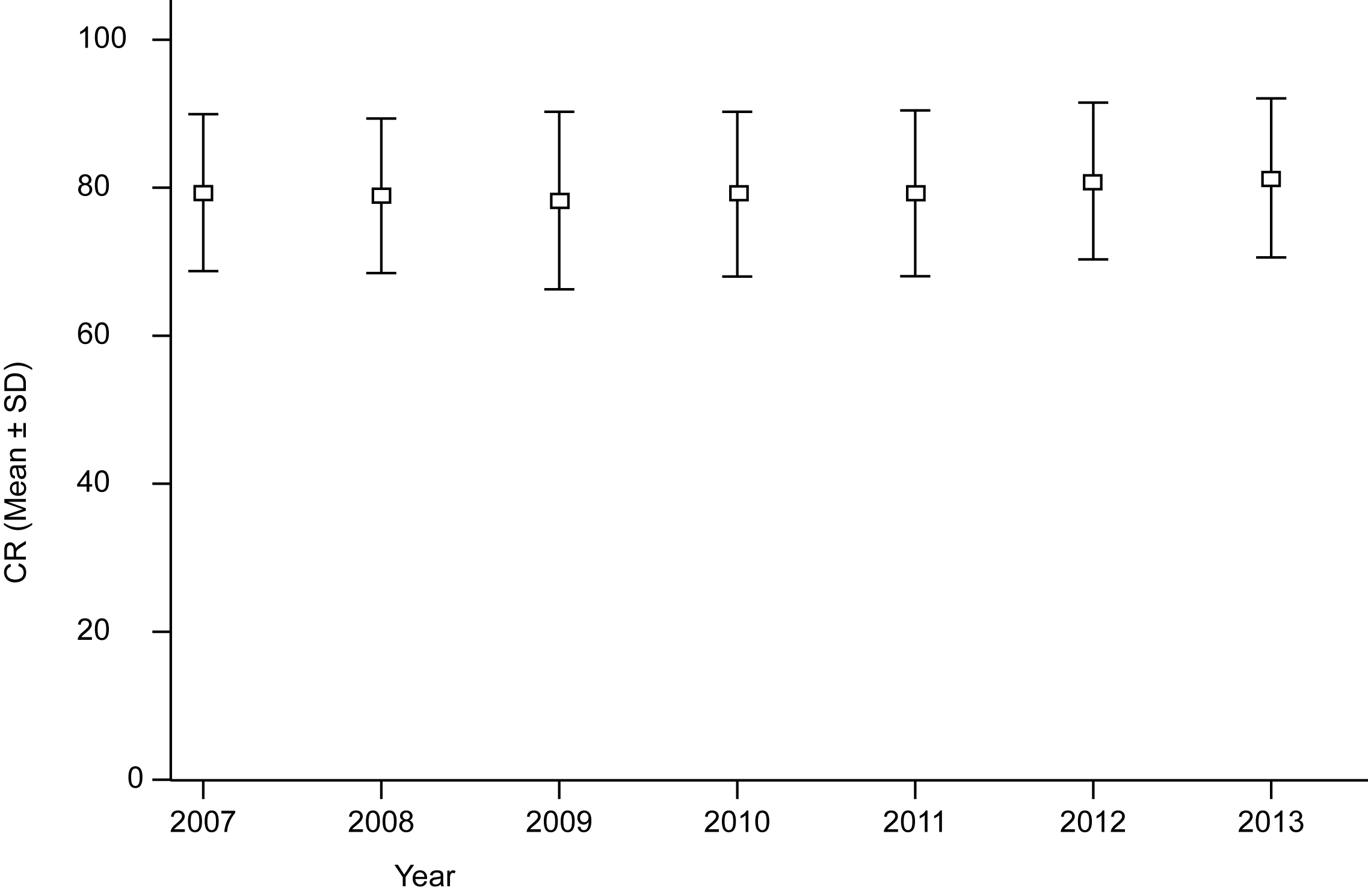
**

**Figure S3.** Distribution of proposals by their differences between Consensus Reports (CR) and average Individual Evaluation Reports (AVIER) scores (N = 24897, mean = –0.3, standard deviation = 3.19).


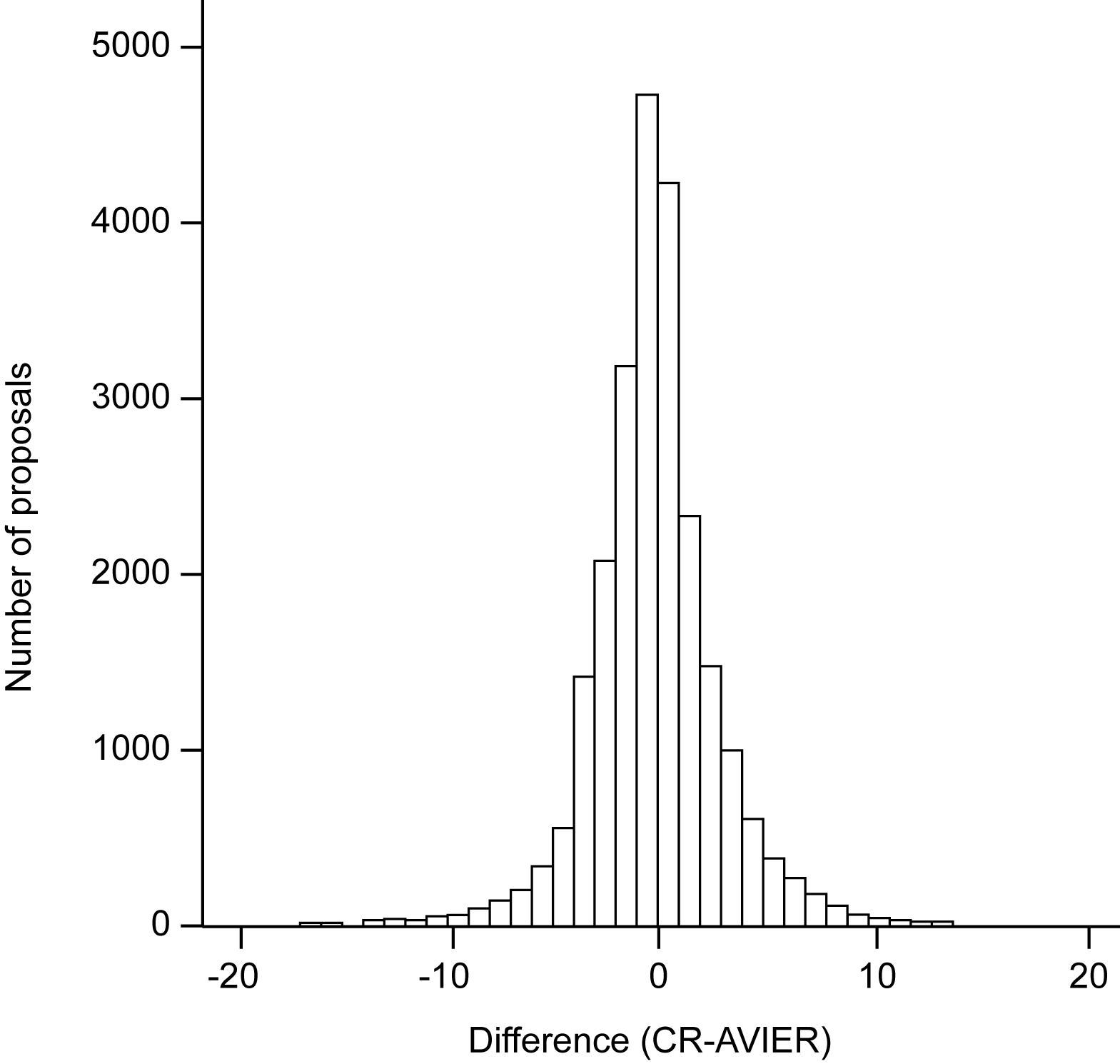


**Table S1.** Inter-rater agreement (average deviation index, AD index) and intraclass correlation coefficients (ICC) for individual evaluation criteria across all evaluation panels (scale 0-5).

|  |  | **AD Index** | | | **ICC^†^** | **ICC* for IER (individual evaluator report)** |
| --- | --- | --- | --- | --- | --- | --- |
| **Panel** | **Criterion** | **Median** | **Quartile 1** | **Quartile 3** |  |  |
| Chemistry | Science and Technology (S&T) quality | 0.33 | 0.20 | 0.51 | 0.52 | 0.64 |
|  | Training (Transfer of Knowledge for IAPP) | 0.36 | 0.22 | 0.53 | 0.62 |  |
|  | Researcher (only for IEF) | 0.22 | 0.16 | 0.36 | 0.55 |  |
|  | Implementation | 0.33 | 0.20 | 0.51 | 0.59 |  |
|  | Impact | 0.31 | 0.20 | 0.51 | 0.57 |  |
| Economy/Social sciences/Humanities | Science and Technology (S&T) quality | 0.44 | 0.27 | 0.64 | 0.53 | 0.65 |
|  | Training (Transfer of Knowledge for IAPP) | 0.44 | 0.29 | 0.67 | 0.61 |  |
|  | Researcher (only for IEF) | 0.31 | 0.20 | 0.47 | 0.52 |  |
|  | Implementation | 0.42 | 0.27 | 0.64 | 0.62 |  |
|  | Impact | 0.42 | 0.27 | 0.64 | 0.59 |  |
| Information Science/Engineering | Science and Technology (S&T) quality | 0.40 | 0.24 | 0.58 | 0.55 | 0.67 |
|  | Training (Transfer of Knowledge for IAPP) | 0.38 | 0.24 | 0.58 | 0.63 |  |
|  | Researcher (only for IEF) | 0.27 | 0.18 | 0.40 | 0.55 |  |
|  | Implementation | 0.38 | 0.24 | 0.56 | 0.64 |  |
|  | Impact | 0.38 | 0.22 | 0.56 | 0.59 |  |
| Environment | Science and Technology (S&T) quality | 0.33 | 0.20 | 0.53 | 0.52 | 0.66 |
|  | Training (Transfer of Knowledge for IAPP) | 0.36 | 0.22 | 0.51 | 0.63 |  |
|  | Researcher (only for IEF) | 0.24 | 0.16 | 0.36 | 0.58 |  |
|  | Implementation | 0.33 | 0.20 | 0.49 | 0.63 |  |
|  | Impact | 0.31 | 0.20 | 0.49 | 0.60 |  |
| Life Sciences | Science and Technology (S&T) quality | 0.31 | 0.20 | 0.49 | 0.56 | 0.68 |
|  | Training (Transfer of Knowledge for IAPP) | 0.33 | 0.22 | 0.51 | 0.64 |  |
|  | Researcher (only for IEF) | 0.22 | 0.16 | 0.36 | 0.57 |  |
|  | Implementation | 0.31 | 0.20 | 0.49 | 0.63 |  |
|  | Impact | 0.33 | 0.20 | 0.49 | 0.63 |  |
| Mathematics | Science and Technology (S&T) quality | 0.33 | 0.20 | 0.53 | 0.52 | 0.63 |
|  | Training (Transfer of Knowledge for IAPP) | 0.38 | 0.24 | 0.58 | 0.62 |  |
|  | Researcher (only for IEF) | 0.27 | 0.18 | 0.40 | 0.46 |  |
|  | Implementation | 0.36 | 0.22 | 0.53 | 0.65 |  |
|  | Impact | 0.38 | 0.22 | 0.56 | 0.60 |  |
| Physics | Science and Technology (S&T) quality | 0.31 | 0.18 | 0.47 | 0.54 | 0.65 |
|  | Training (Transfer of Knowledge for IAPP) | 0.33 | 0.22 | 0.51 | 0.59 |  |
|  | Researcher (only for IEF) | 0.22 | 0.13 | 0.33 | 0.61 |  |
|  | Implementation | 0.31 | 0.18 | 0.47 | 0.62 |  |
|  | Impact | 0.31 | 0.20 | 0.47 | 0.61 |  |

*One-way random intraclass correlation coefficient (ICC_1_) for average measures; this is an appropriate measure of average inter-rater agreement when raters are not the same for all items (i.e. different raters rate different items)

**Table S2.** Distribution of proposals, across panels and type of action, where a) one rater disagrees with other two raters; b) all raters disagree with each other; c) difference between the Consensus Report (CR) and average Individual Evaluation Report (AVIER) score is large.

| **Call** | **Panel** | **Disagreement (No. proposals, row %)** | | |
| --- | --- | --- | --- | --- |
|  |  | One rater differs* | All raters differ† | AVIER vs CR difference‡ |
| **IAPP** | Chemistry (n=63) | 5 (7.9%) | 9 (14.3%) | 2 (3.2%) |
|  | Economic Sciences/Social Sciences/Humanities (n=68) | 6 (8.8%) | 10 (14.7%) | 4 (5.9%) |
|  | Information Science/Engineering (n=296) | 30 (10.1%) | 41 (13.9%) | 7 (2.4%) |
|  | Environment (n=84) | 10 (11.9%) | 19 (22.6%) | 3 (3.6%) |
|  | Life Sciences (n=203) | 18 (8.9%) | 37 (18.2%) | 7 (3.4%) |
|  | Mathematics (n=6) | 0 (0.0%) | 3 (50%) | 0 (0.0%) |
|  | Physics (n=39) | 2 (5.1%) | 5 (12.8%) | 0 (0.0%) |
|  | **Total (n=759)** | **71 (9.4%)** | **124 (16.3%)** | **23 (3.0%)** |
| **IEF** | Chemistry (n=2204) | 93 (4.2%) | 118 (5.4%) | 12 (0.5%) |
|  | Economic Sciences/Social Sciences/Humanities (n=4228) | 387 (9.2%) | 527 (12.5%) | 119 (2.8%) |
|  | Information Science/Engineering (n=1888) | 103 (5.5%) | 172 (9.1%) | 23 (1.2%) |
|  | Environment (n=2731) | 116 (4.2%) | 172 (6.3%) | 27 (1.0%) |
|  | Life Sciences (n=6408) | 255 (4.0%) | 354 (5.5%) | 43 (0.7%) |
|  | Mathematics (n=665) | 35 (5.3%) | 54 (8.1%) | 4 (0.6%) |
|  | Physics (n=2469) | 84 (3.4%) | 139 (5.6%) | 13 (0.5%) |
|  | **Total (n=20593)** | **1073 (5.2%)** | **1536 (7.5%)** | **241 (1.2%)** |
| **ITN** | Chemistry (n=398) | 34 (8.5%) | 44 (11.1%) | 18 (4.5%) |
|  | Economic Sciences/Social Sciences/Humanities (n=381) | 38 (10.0) | 63 (16.5%) | 19 (5.0%) |
|  | Information Science/Engineering (n=799) | 66 (8.3%) | 93 (11.3%) | 20 (2.5%) |
|  | Environment (n=428) | 27 (6.3%) | 39 (9.1%) | 12 (2.8%) |
|  | Life Sciences (n=1047) | 81 (7.7%) | 128 (12.2%) | 21 (2.0%) |
|  | Mathematics (n=60) | 6 (10.0%) | 10 (16.7%) | 1 (1.7%) |
|  | Physics (n=432) | 28 (6.5%) | 38 (8.8%) | 13 (3.0%) |
|  | **Total (n=3545)** | **280 (7.9%)** | **415 (11.7%)** | **104 (2.9%)** |

*Disagreement is defined as one rater differing 10 or more points from other two raters, who agree within 5 points on a scale from 0 to100.

†Disagreement is defined as all raters differing 10 or more points from each other on a scale from 0 to100.

‡Disagreement is defined as equal or greater than 10 points on a scale from 0 to100.

**Table S3.** Pearson’s correlations between IER and CR scores for separate criteria*

|  | | | **Consensus Report** | | | | |
| --- | --- | --- | --- | --- | --- | --- | --- |
|  |  |  | **S&T quality** | **Training/ToK** | **Researcher** | **Implementation** | **Impact** |
| **Individual Evaluation Report** | **S&T quality** | Rater 1 | 0.678 | 0.534 | 0.476 | 0.522 | 0.529 |
|  |  | Rater 2 | 0.678 | 0.533 | 0.469 | 0.522 | 0.521 |
|  |  | Rater 3 | 0.681 | 0.523 | 0.468 | 0.515 | 0.529 |
|  | **Training/ToK** | Rater 1 | 0.546 | 0.713 | 0.471 | 0.578 | 0.588 |
|  |  | Rater 2 | 0.549 | 0.715 | 0.468 | 0.584 | 0.587 |
|  |  | Rater 3 | 0.553 | 0.705 | 0.463 | 0.578 | 0.588 |
|  | **Researcher** | Rater 1 | 0.458 | 0.448 | 0.671 | 0.447 | 0.484 |
|  |  | Rater 2 | 0.459 | 0.457 | 0.662 | 0.445 | 0.484 |
|  |  | Rater 3 | 0.469 | 0.451 | 0.656 | 0.454 | 0.497 |
|  | **Implementation** | Rater 1 | 0.538 | 0.585 | 0.528 | 0.694 | 0.595 |
|  |  | Rater 2 | 0.537 | 0.576 | 0.514 | 0.690 | 0.581 |
|  |  | Rater 3 | 0.541 | 0.572 | 0.516 | 0.688 | 0.582 |
|  | **Impact** | Rater 1 | 0.544 | 0.588 | 0.512 | 0.599 | 0.695 |
|  |  | Rater 2 | 0.538 | 0.583 | 0.507 | 0.598 | 0.692 |
|  |  | Rater 3 | 0.542 | 0.575 | 0.501 | 0.594 | 0.691 |

*Evaluation criteria: Science and Technology (S&T) quality; Training (for ITN and IEF) or Transfer of Knowledge (ToK, for IAPP); Researcher (criterion used only for IEF); Implementation; Impact. N = 24897 for all except for “Researcher” where N = 20593. All correlations were statistically significant at P<0.001 level.
